# Transfusion-Related Iron Overload in Children With Acute Lymphoblastic Leukemia and Lymphoblastic Lymphoma: Addressing an Overlooked Treatment Complication

**DOI:** 10.7759/cureus.103334

**Published:** 2026-02-10

**Authors:** Nusa Matijasic Stjepovic, Izabela Kranjcec, Arnes Resic, Sara Sila, Ana Cavar, Lucija Ruzman, Elizabeta Trbusic, Jasminka Stepan Giljevic

**Affiliations:** 1 Department of Oncology and Hematology, Children's Hospital Zagreb, Zagreb, HRV; 2 Department of Pediatrics, Faculty of Medicine, University of Rijeka, Rijeka, HRV; 3 Department of Pediatrics, University North, Varazdin, HRV; 4 Department of Pediatric Clinical Pharmacology and Toxicology, Children’s Hospital Zagreb, Zagreb, HRV; 5 University Department of Health Studies, University of Split, Split, HRV; 6 Referral Centre for Pediatric Gastroenterology and Nutrition, Children's Hospital Zagreb, Zagreb, HRV; 7 Department of Blood Transfusion, Croatian Institute of Transfusion Medicine, Zagreb, HRV; 8 Department of Pediatrics, Division of Hematology, Oncology and Clinical Genetics, Clinical Hospital Centre Rijeka, Rijeka, HRV; 9 Pediatric Primary Care Department, Health Center Zagreb, Zagreb, HRV; 10 School of Medicine, University of Zagreb, Zagreb, HRV

**Keywords:** deferasirox, ferritin, hyperferritinemia, iron chelation therapy, iron overload, pediatric acute lymphoblastic leukemia, pediatric lymphoblastic lymphoma, secondary hemochromatosis, trasfusion-related iron overload

## Abstract

Introduction: Transfusion-related iron overload (TRIO) is a common antineoplastic treatment complication in children undergoing aggressive chemotherapy for hematological malignancies. Iron deposition leads to numerous morbidities, with the most devastating outcomes being liver and heart failure.

Materials and methods: An observational retrospective study on TRIO was performed on children with acute lymphoblastic leukemia (ALL) and lymphoblastic lymphoma (LL) treated at the Department of Oncology and Hematology, Children's Hospital Zagreb, Croatia, from January 1, 2018, to December 31, 2023. Epidemiological and basic clinical data were retrieved from the patients’ electronic medical records. Serum ferritin (SF) concentration (ng/mL) was used as a marker of TRIO. Mildly elevated ferritin was defined as SF >500 ng/mL and severely elevated as SF >1000 ng/mL. Results of magnetic resonance imaging (MRI) studies for tissue iron quantification, *HFE* genetic analyses, and management with chelators (preparation, dose, duration, compliance) were described.

Results: Initial SF concentrations ranged from 17.5 to 808 ng/mL, rose to the range from 368 to 6562.3 ng/mL at the end of the intensive chemotherapy, sunk to the range from 11.9 to 3885 ng/mL at the end of the maintenance therapy, and additionally receded to the range from 36.2 to 1924 ng/mL during the follow-up (FU). Upon cessation of intensive chemotherapy, significant hyperferritinemia was detected in 96% of the patients tested and during the FU in 60% of them. Risk factors for TRIO included age six and older, high-risk (HR) disease, and a substantial transfusion load (≥10 red blood cell transfusions (RBCTs)). Hepatic and/or cardiac MRI, as part of the TRIO work-up, was performed in only four patients. *HFE* genotyping was conducted in five participants, and the results were altered in three of them - H63D heterozygosity. Four children received chelation therapy, i.e., deferasirox, which was discontinued in two of them due to gastrointestinal discomfort.

Conclusion: We have demonstrated a high occurrence but an unsatisfactory approach to TRIO. A fair number of patients lacked SF measurements during the treatment and FU. Despite a considerable proportion of patients at risk of TRIO in our cohort, only four underwent MRI for tissue iron quantification. Unlike the ferritin assay, MRI is neither accessible nor cheap, and the need for sedation/anesthesia in young children probably leads to underutilization of the method. Furthermore, there was no consensus on genotyping for hereditary hemochromatosis. Older age and HR disease posed a greater risk for TRIO. Transfusion burden significantly impacted ferritin levels. We advise clinicians to follow a restrictive transfusion strategy and track the number of red blood cell units administered throughout the treatment to identify patients with especially worrisome iron influx. There is a strong need for the development of TRIO treatment guidelines for patients and survivors of childhood cancer.

## Introduction

Transfusion-related iron overload (TRIO) is an acquired condition resulting from multiple red blood cell transfusions (RBCTs) given to treat anemias, excluding those caused by iron deficiency or blood loss. It is becoming an increasingly recognized complication in children undergoing antineoplastic treatment [1-12]. Each unit of transfused blood delivers approximately 200-250 mg of iron [13], far exceeding the body’s daily iron elimination capacity, which is about 1-2 mg [14]. Therefore, while lifesaving, RBCTs can rapidly lead to excessive iron deposition, posing a risk of organ damage, primarily through oxidative stress. Commonly related morbidities include endocrinopathies such as hypothyroidism, hypoparathyroidism, growth hormone deficiency, delayed puberty, diabetes, and reduced bone mineral density [8, 15]. The uncontrolled accumulation of iron in hepatocytes and cardiomyocytes results in the most devastating outcomes - liver and heart failure [16].

Recipients of repeated RBCTs are generally monitored using serum ferritin (SF) concentrations and hepatic and cardiac magnetic resonance imaging (MRI) [17,18]. Early detection of TRIO enables timely intervention and prevention of detrimental sequelae. Chelation therapy, designed to eliminate excessive body iron, has become a crucial component of the treatment regimen. It requires thoughtful planning, including its initiation, intensity, and length, especially in coordination with the different stages of chemotherapy [7,13,19].

Within the framework of acute lymphoblastic leukemia (ALL) and lymphoblastic lymphoma (LL), TRIO poses a considerable clinical challenge as signs and symptoms often overlap with those caused by malignancy and numerous other side effects of the harsh and prolonged treatment. Laboratory screening can be difficult to interpret - elevated SF also indicates uncontrolled tumor growth or infection [20]. Moreover, data on TRIO therapeutic management in pediatric oncology are practically nonexistent.

The primary endpoints of this retrospective study were to examine ferritin trends in children during and following aggressive treatments for ALL and LL, and to determine the occurrence of TRIO in this specific pediatric patient group. In addition, the goal was to identify risk factors associated with TRIO, as well as to reveal the overall practice of a tertiary care center concerning iatrogenic iron deposition, including the assessment of organ damage through hepatic and cardiac MRI, genetic testing for hereditary hemochromatosis (HH), and the administration of chelating agents. Most other priorly published work on the subject addressed only certain aspects of the problem, focusing dominantly on factors predicting secondary iron overload (IO). The authors’ goal was to raise awareness of TRIO and hopefully prompt clinicians to monitor for iron toxicities during chemotherapy and survivorship follow-up (FU) care.

## Materials and methods

An observational retrospective study on TRIO was performed at the Department of Oncology and Hematology, Children's Hospital Zagreb, Croatia, from January 1, 2018, to December 31, 2023. Inclusion criteria for children of both sexes were age 0-18 years and the diagnosis of ALL and LL. Exclusion criteria were previously known oncological, benign hematological, or any other conditions potentially contributing to IO. 

Epidemiological (age, sex, and year of diagnosis) and basic clinical data (diagnosis, malignant immunophenotype, risk group/stage, treatment protocol, transplantation, and outcome) were retrieved from the patients’ electronic medical records.

SF concentration (ng/mL) was used as the laboratory marker of TRIO. All SF measurements were performed at the biochemical laboratory of the Children’s Hospital Zagreb, using the chemiluminescence immunoassay. SF concentrations analyzed were those measured at four time points of interest, depending on availability, given the retrospective study design. The designated timestamps were the following: T1 - at the time of diagnosis/baseline, T2 - at the end of the intensive treatment, T3 - at the end of the maintenance therapy, and T4 - during the last FU visit at which SF was quantified. The cut-off for mildly elevated SF was set to >500 ng/mL, and for severely elevated SF, it was >1000 ng/mL, following the example of a similar preexisting study [1].

Differences in SF levels at T1-T4 depending on sex, age, diagnosis, malignant immunophenotype, and ALL risk group were tested. Participants were divided into two age groups (<6 and ≥6 years) based on the median age, which was six. Moreover, SF levels were compared for age groups younger than 10 and 10 and older, as, based on the available literature, pediatric patients older than 10 are at a greater risk of developing TRIO [1,4]. Patients with ALL were assigned a risk group (standard-risk - SR, intermediate-risk - IR, or high-risk - HR) according to the International Berlin-Frankfurt-Münster Study Group Protocol for ALL (ALL IC-BFM). Depending on the extent of the disease, patients with LL were assigned a corresponding stage (I-IV).

The number of red blood cell (RBC) packs administered (units) was written down, and the volume received by each patient (mL/kg) was calculated based on the patient’s body mass recorded at diagnosis (T1). According to the treatment protocol, the hemoglobin (Hb) threshold for blood transfusion was 8 g/dL. In exceptional circumstances of symptomatic anemia and conditions that aggravate tissue oxygenation (hemorrhage, severe infection), RBC packs were delivered regardless of Hb concentration exceeding the threshold. When investigating the correlation between ferritin and the number of RBC packs delivered, patients were divided into those who received <10 and ≥10 RBC units, a quantity previously identified as contributory to IO [3,4,7].

Results of the MRI studies, performed as part of the TRIO work-up, were described. Iron chelation therapy (preparation, dose, duration, and compliance) was noted, as well as the findings of genetic testing for HH (*HFE* genotype), when conducted.

The normality of the data was assessed by the Shapiro-Wilk test. Numerical data were presented as median (min-max) and categorical data as N (%). The differences for non-categorical data were assessed using the Mann-Whitney U test or the Kruskal-Wallis test, and pairwise comparisons were performed when applicable. Significant values have been adjusted by the Bonferroni correction for multiple tests. A p-value of 0.05 was considered statistically significant in a two-sided test.

The survey was conducted according to the Declaration of Helsinki and approved by the Institutional Ethical Board of Children's Hospital Zagreb (approval No. 01-23/5-5-24, 1.2.2024)

## Results

Forty children (mean age 5.5 years, median six years), with a slight female predominance (N = 22, 55%), treated for ALL and LL at the center during the six-year period, met the inclusion criteria. The majority of patients (N = 11) were diagnosed in 2018. Most leukemia patients were treated according to the ALL-IC BFM protocol (in one infant, the Interfant-06 protocol was applied), while the EURO-LB 2002 protocol was used in all lymphoma cases. Disease characteristics and outcomes are depicted in Table 1. 

**Table 1:** Disease characteristics, type of hematopoietic stem cell transplantation (if performed), and outcomes The table depicts disease characteristics, including diagnosis, malignant immunophenotype, ALL risk group, LL stage, type of HSCT (if performed), and outcomes. ALL: acute lymphoblastic leukemia, HSCT: hematopoietic stem cell transplantation, LL: lymphoblastic lymphoma, N: total number, Ph-like: Philadelphia-like ALL, a subset of high-risk leukemias with different molecular alterations

Disease characteristics and outcomes	N	%
Diagnosis		
ALL	35	87.5
- Ph-like ALL	1	2.5
- Infant ALL	1	2.5
LL	5	12.5
Immunophenotype		
B	34	85
T	6	15
ALL risk group (N, 35)		
Standard	5	14.3
Intermediate	19	54.3
High	11	31.4
LL Stage (N, 5)		
I	1	20
IV	4	80
HSCT		
Autologous	1	2.5
Allogeneic	2	5
Outcome		
Deceased	3	7.5
Survived	37	92.5

Out of 40 subjects, 37 (92.5%) had SF evaluated at baseline (T1), revealing concentrations above 500 ng/mL in three of them (8.1%), while none had SF above 1000 ng/mL. Upon cessation of the intensive chemotherapy phase (T2), SF concentrations were obtained in 25 patients (62.5%), demonstrating mild hyperferritinemia (SF 501-1000 ng/mL) in three patients (12%), and severe hyperferritinemia (SF >1000 ng/mL) in 21 of them (84%). Three patients died during the treatment, and only 37 reached the T3-T4 time points. At the end of the maintenance phase (T3), ferritin was measured in 20 out of those 37 subjects (54.1%), showing mildly elevated levels in 9 (45%), and severely elevated in 6 of them (30%). During the FU (T4), SF was tested in 30 out of 37 survivors (81.1%); it ranged from 501 to 1000 ng/mL in 12 of them (40%) and exceeded 1000 ng/mL in six of them (20%). 

**Figure 1:**
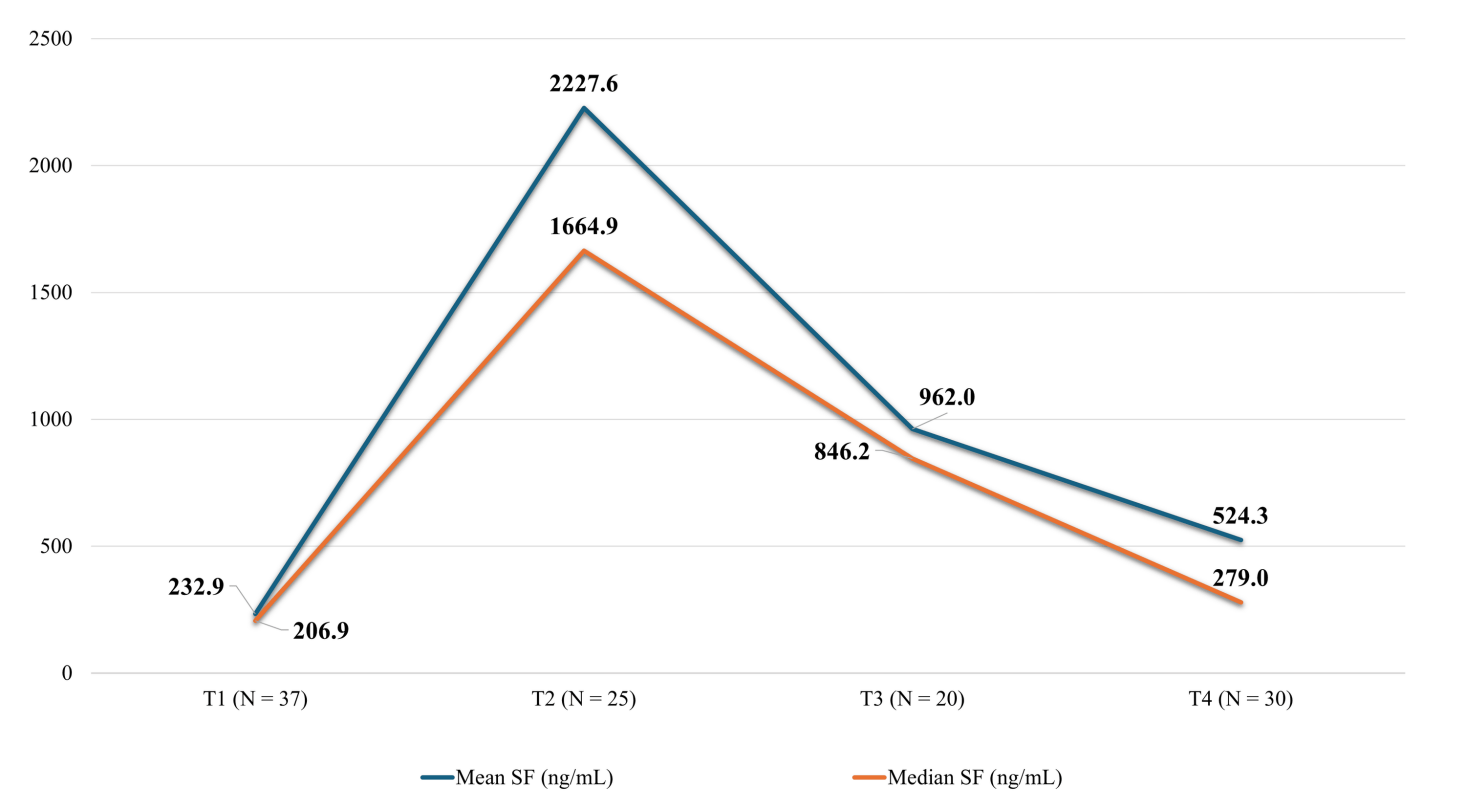
Dynamics of serum ferritin concentrations at diagnosis, at the end of the intensive and maintenance therapy phase and during the follow-up Serum ferritin concentrations (represented as mean and median) rose from their baseline values at diagnosis (T1), peaked at the end of the intensive therapy phase (T2), and, despite a decline at the end of the maintenance phase (T3), remained above pediatric reference ranges during the follow-up period (T4). N: total number of participants with obtained ferritin values at different time points, SF: serum ferritin, T1: first time point - at diagnosis, T2: second time point - at the end of the intensive therapy phase, T3: third time point - at the end of the maintenance phase, T4: fourth time point - during the follow-up

Initial (T1) SF concentrations ranged from 17.5 to 808 ng/mL, rose to the range from 368 to 6562.3 ng/mL at the end of the intensive chemotherapy (T2), sunk to the range from 11.9 to 3885 ng/mL at the end of the maintenance therapy (T3), and additionally receded to the range from 36.2 to 1924 ng/mL during the FU (T4). The patient with the highest starting ferritin value (808 ng/mL) was a teenage male diagnosed with stage IV LL. The value of 6562.3 ng/mL, measured at T2, represented at the same time the maximal SF level recorded in our cohort. It was detected in an eight-year-old female patient with HR B-ALL, significantly burdened with repeated transfusions (17 RBC packs, 139.1 mL/kg). Dynamics of SF levels at T1-T4 are presented in Figure 1. 

No significant differences in ferritin concentrations were determined at all time points (T1-4) regarding sex (male vs. female), diagnosis (ALL vs. LL), or immunophenotype (B vs. T). HR patients had significantly higher SF values at the end of the intensive and maintenance phase (T2, T3), compared to SR patients. The same - significantly higher SF in HR patients - was noted at the end of the intensive treatment (T2) and during the FU (T4), when compared to the IR group. Children aged six and older had significantly higher SF measurements at the end of both the intensive and maintenance phase (T2, T3), compared to younger kids. No such difference was observed when the age limit of 10 years was set. However, this observation must be carefully interpreted due to statistical weakness, as only four subjects were 10 or older at the time of diagnosis.Tables 2-7 summarize the study’s results, showing mean and median SF concentrations sorted by categories.

**Table 2:** Mean and median serum ferritin concentrations at different time points (T1-T4) according to sex (male vs. female participants) The table summarizes the study’s results, showing mean and median SF concentrations at different time points (T1-T4), compared by sex (male vs. female participants). Analysis was performed using the Mann-Whitney U test (independent samples). SF: serum ferritin, T1: first time point - at diagnosis, T2: second time point - at the end of the intensive therapy phase, T3: third time point - at the end of the maintenance phase, T4: fourth time point - during the follow-up

Time point	Mean SF (ng/mL) *Male *		Mean SF (ng/mL) *Female *		Median SF (ng/mL) *Male *		Median SF (ng/mL) *Female *		p-value			Mann-Whitney U	Z
														T1	278.0		194.7		226.8		170.2		0.220			129.000	-1.250
														T2	2314.4		2169.8		1871.4		1415.1		0.367			58.000	-0.940
														T3	1088.4		835.6		852.2		754.1		0.631			43.000	-0.530
T4	505.2		543.4		183.5		418.8		0.436			93.000	-0.810

**Table 3:** Mean and median serum ferritin concentrations at different time points (T1-T4) according to age (<6 vs. ≥6 years) The table summarizes the study’s results, showing mean and median SF concentrations at different time points (T1-T4), compared by age (patients younger than six vs. six and older). Analysis was performed using the Mann-Whitney U test (independent samples). SF: serum ferritin, T1: first time point - at diagnosis, T2: second time point - at the end of the intensive therapy phase, T3: third time point - at the end of the maintenance phase, T4: fourth time point - during the follow-up

Time point	Mean SF (ng/mL) *Age <6 years*		Mean SF (ng/mL) *Age ≥6 years*		Median SF (ng/mL) *Age <6 years*		Median SF (ng/mL) *Age ≥6 years*		p-value			Mann-Whitney U	Z
														T1	242.4		219.1		203.0		249.0		0.867			159.000	-0.190
														T2	1746.1		2949.9		1381.9		2462.9		0.031			36.000	-2.163
														T3	567.5		1694.6		568.0		1267.8		0.000			4.000	-3.289
T4	419.9		704.7		177.2		753.0		0.064			61.000	-1.872

**Table 4:** Mean and median serum ferritin concentrations at different time points (T1-T4) according to age (<10 vs. ≥10 years) The table summarizes the study’s results, showing mean and median SF concentrations at different time points (T1-T4), compared by age (patients younger than 10 vs. 10 and older). Analysis was performed using the Mann-Whitney U test (independent samples). * Values could not be calculated as only one measurement in the specific group was obtained. SF: serum ferritin, T1: first time point - at diagnosis, T2: second time point - at the end of the intensive therapy phase, T3: third time point - at the end of the maintenance phase, T4: fourth time point - during the follow-up

Time point	Mean SF (ng/mL) *Age <10 years*		Mean SF (ng/mL)Age ≥10 years		Median SF (ng/mL) *Age <10 years*		Median SF (ng/mL) *Age ≥10 years*		p-value			Mann-Whitney U	Z
														T1	336.6		248.0		226.8		262.0		0.957			46.000	-0.980
														T2	2208.5		2367.3		1427.0		2189.6		0.398			16.000	-0.700
														T3	945.9		*		835.3		*		*			*	*
														T4	492.3		812.3		209.2		1026.8		0.155			19.000	-1.486

**Table 5:** Mean and median serum ferritin concentrations at different time points (T1-T4) according to diagnosis (acute lymphoblastic leukemia vs. lymphoblastic lymphoma) The table summarizes the study’s results, showing mean and median SF concentrations at different time points (T1-T4), compared by diagnosis (ALL vs. LL). Analysis was performed using the Mann-Whitney U test (independent samples). * Values could not be calculated as only one measurement in the specific group was obtained. ALL: acute lymphoblastic leukemia, LL: lymphoblastic lymphoma, SF: serum ferritin, T1: first time point - at diagnosis, T2: second time point - at the end of the intensive therapy phase, T3: third time point - at the end of the maintenance phase, T4: fourth time point - during the follow-up

Time point	Mean SF (ng/mL) *ALL*		Mean SF (ng/mL) *LL*		Median SF (ng/mL) *ALL*		Median SF (ng/mL) *LL*		p-value			Mann-Whitney U	Z
														T1	232.6		235.4		226.8		58.1		0.203			39.000	-1.321
														T2	2122.2		3000.5		1551.9		3713.2		0.273			19.000	-1.171
														T3	928.3		*		835.3		*		*			*	*
T4	480.4		809.7		255.5		774.3		0.391			37.000	-0.195

**Table 6:** Mean and median serum ferritin concentrations at different time points (T1-T4) according to immunophenotype (B vs. T) The table summarizes the study’s results, showing mean and median SF concentrations at different time points (T1-T4), compared by immunophenotype (B vs. T). Analysis was performed using the Mann-Whitney U test (independent samples). SF: serum ferritin, T1: first time point - at diagnosis, T2: second time point - at the end of the intensive therapy phase, T3: third time point - at the end of the maintenance phase, T4: fourth time point - during the follow-up

Time point	Mean SF (ng/mL) *B immunophenotype*		Mean SF (ng/mL) *T immunophenotype*		Median SF (ng/mL) *B immunophenotype*		Median SF (ng/mL) *T immunophenotype*		p-value			Mann-Whitney U	Z
														T1	241.2		180.2		216.9		30.2		0.307			56.000	-1.066
														T2	2212.7		2399.6		1664.9		2399.6		1.000			23.000	0.000
														T3	954.8		1002.4		835.3		857.1		0.765			22.000	-0.370
T4	515.8		579.5		279.0		314.0		0.883			49.000	-0.180

**Table 7:** Mean and median serum ferritin concentrations at different time points (T1-T4) according to the acute lymphoblastic leukemia risk group The table summarizes the study’s results, showing mean and median SF concentrations at different time points (T1-T4), compared by the ALL risk group (IR vs. SR vs. HR). Analysis was performed using the Mann-Whitney U test, adjusted by the Bonferroni correction for multiple tests. ALL: acute lymphoblastic leukemia, HR: high-risk, IR: intermediate-risk, LL: lymphoblastic lymphoma, SF: serum ferritin, SR: standard-risk, T1: first time point - at diagnosis, T2: second time point - at the end of the intensive therapy phase, T3: third time point - at the end of the maintenance phase, T4: fourth time point - during the follow-up

Time point	Mean SF (ng/mL) *SR ALL*	Mean SF (ng/mL) *IR ALL*		Mean SF (ng/mL) *HR ALL*	Median SF (ng/mL) *SR ALL*	Median SF (ng/mL) *IR ALL*		Median SF (ng/mL) *HR ALL*	p-value *SR/IR*	p-value *SR/HR*	p-value *IR/HR*
												T1	367.6	219.3		204.1	384.7	199.0		199.5	0.044	0.142	0.636
												T2	1045.4	1506.6		3704.9	950.8	1229.8		2528.6	0.916	0.010	0.030
												T3	511.3	677.6		2034.6	491.9	835.3		1698.1	0.999	0.030	0.094
T4	248.7	388.8		829.2	104.4	158.6		913.8	1.000	0.058	0.037

**Figure 2:**
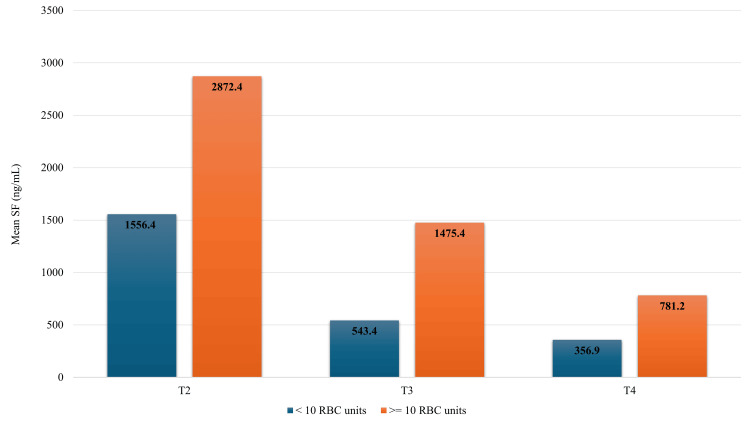
Mean serum ferritin concentrations at different time points (T2-T4) depending on the number of red blood cell units transfused Patients with a greater transfusion burden (≥10 RBC units) had significantly higher mean SF concentrations during and after antineoplastic treatment. RBC: red blood cell, SF: serum ferritin, T2: second time point - at the end of the intensive therapy phase, T3: third time point - at the end of the maintenance phase, T4: fourth time point - during the follow-up

The median number of RBC packs administered during the treatment in our population was 10 units (mean 11.1, range 1-43), while the median volume calculated according to the patient's body mass was 89.5 mL/kg (mean 94.3 mL/kg, range 11.4-212.6 mL/kg). The mean number of RBC packs received by SR and IR ALL patients was 7.9, while it was 18.1 received by HR ALL patients. The mean transfusion volume according to the patient’s body mass was 84 mL/kg in the combined SR and IR ALL group, and 135 mL/kg in the HR group. The highest number of transfusions, 43, was administered to a 14-year-old male patient with HR B-ALL. He finished each treatment phase with severely elevated SF, and his SF at FU also exceeded 1000 ng/mL. A total of 19 patients (47.5%) received ≥10 RBC units. This group had significantly higher SF at T2-T4, compared to the group of patients who received less than 10 transfusions (T2 p = 0.006; T3 p = 0.006; T4 p = 0.025). Figure 2 illustrates the influence of transfusion load on ferritin levels during and after the treatment ended.

MRI of organs at risk (liver/heart) was done as a part of IO monitoring in only four patients. MRI findings and relevant clinical information in the above-mentioned cases are summed up in Table 8.

**Table 8:** MRI findings in patients in whom imaging was done as part of the transfusion-related iron overload work-up The table represents MRI findings in patients who underwent imaging as part of the TRIO work-up. It additionally explains why the imaging was indicated, contains information on maximal SF measured in these patients, the amount of RBCTs administered (units and volume), as well as the rationale for starting chelation therapy. ALL: acute lymphoblastic leukemia, F: female, FU: follow-up, HR: high-risk, HSCT: hematopoietic stem cell transplantation, IO: iron overload, LL: lymphoblastic lymphoma, M: male, Max.: maximal, MRI: magnetic resonance imaging, RBCT: red blood cell transfusion, SF: serum ferritin, TRIO: transfusion-related iron overload

Patient	Diagnosis	Organs screened	Indication	MRI findings	Max. SF (ng/mL)	RBCTs (units)	RBCTs (mL/kg)	Chelation therapy (Yes/No)	Rationale for treatment initiation
1. M, 13 years	stage IV B-LL	Liver and heart	SF persistently >1000 ng/mL during the FU, cardiomyopathy	Without signs of IO	1435.9	8	40.5	Yes	Severe hyperferritinemia during the FU, potential aggravation of anthracycline cardiomyopathy
2. F, 5 years	HR B-ALL	Liver	SF persistently >5000 ng/mL during the FU, high transfusion burden following allogeneic HSCT	Without signs of IO	5263.9	13	113	No	/
3. M, 14 years	HR B-ALL	Brain, liver and heart	*Brain* - psychosis, convulsions, final diagnosis: steroid-induced psychosis; *Liver* and *heart *- suspicion of IO following accidental findings on brain MRI	*Brain* - findings indicative of IO: signal decline in the gradient-echo sequences visible in the choroid plexuses of the lateral ventricles, the IV ventricle, and in part of the cerebrospinal fluid space of the left cerebellar hemisphere; *Liver* - voluminous with T2 relaxation time of 3.3 ms, indicating moderate iron deposition; *Heart* - without signs of IO	4001.1	43	212.4	Yes	Severe hyperferritinemia, high transfusion burden, MRI confirmation of secondary brain and liver IO
4. M, 16 years	HR T-ALL	Brain and liver	*Brain* - somnolence nausea, vomiting, final diagnosis: complications following HR blocks - mucositis, dehydration and electrolyte disbalance; *Liver* - suspicion of IO following accidental findings on brain MRI	*Brain *- findings indicative of IO: signal decline in the gradient-echo sequences visible in the choroid plexuses of the lateral ventricles and the IV ventricle; *Liver* - without signs of IO	2934.5	33	157.6	Yes	Severe hyperferritinemia, high transfusion burden, MRI confirmation of secondary brain IO

**Figure 3:**
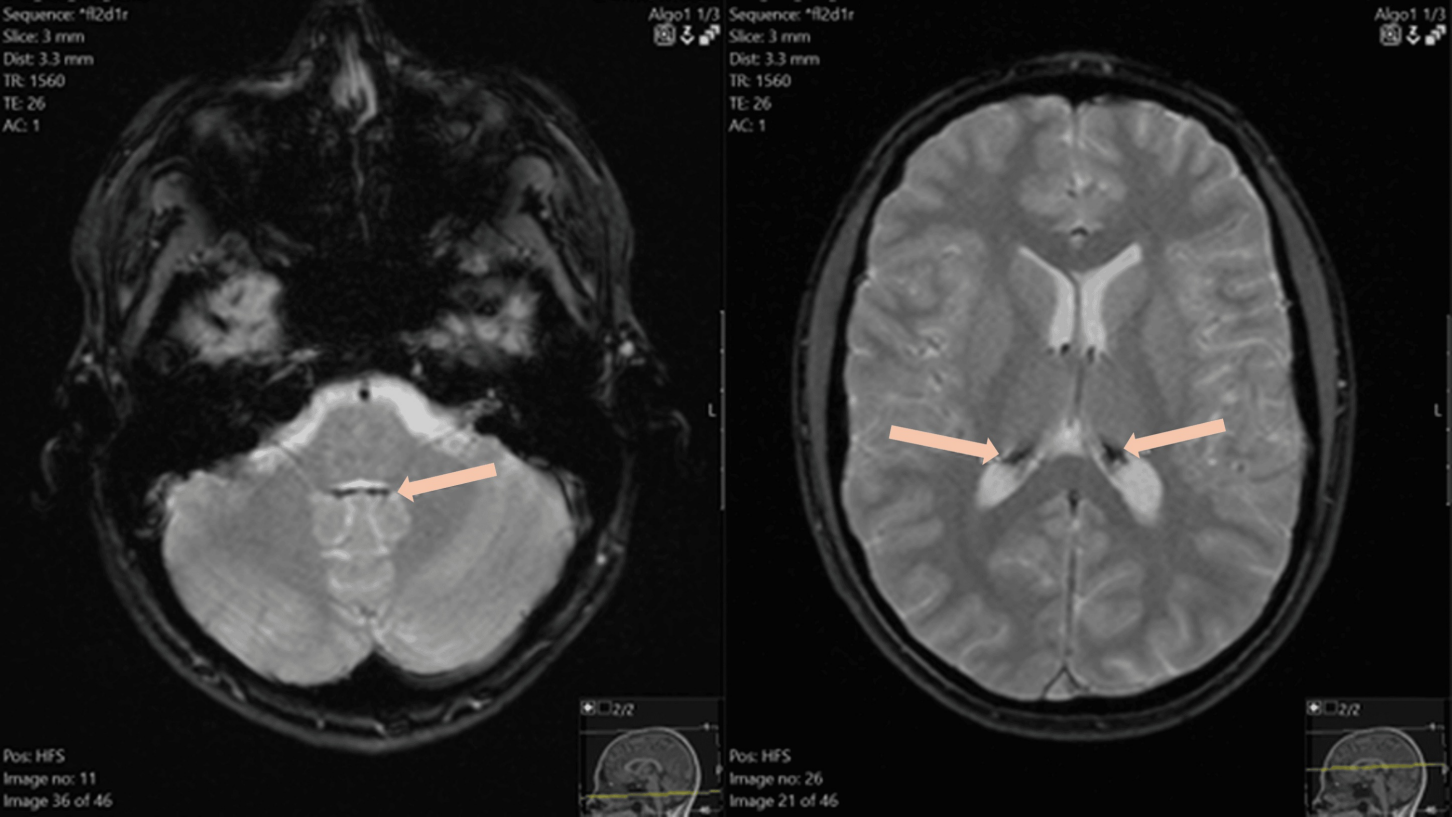
Brain MRI of a 14-year-old male with HR B-ALL and secondary cerebral iron deposits Figure displays MRI findings indicative of brain IO - signal decline visible in the choroid plexuses of the lateral ventricles and the IV ventricle. ALL: acute lymphoblastic leukemia, HR: high-risk, IO: iron overload, MRI: magnetic resonance imaging

Figure 3 displays a brain MRI of a teenage boy with incidental findings of cerebral tissue IO. 

*HFE* genotyping was performed in five participants, and the results were altered in three of them - H63D heterozygosity. All four patients depicted in Table 3 underwent genotyping, and the fifth one was a 14-year-old male with IR ALL who received a substantial amount of RBC units and had severe hyperferritinemia recorded repeatedly during the FU, without clinical signs of TRIO.

In four children, iron chelation therapy was initiated, the preferred medicine being deferasirox in the formulation of dispersible tablets. Three of those patients are presented in Table 3. The fourth one was a girl with HR B-ALL and severe hyperferritinemia following HR blocks, mentioned above as the patient having the maximal SF concentration recorded during the research (6562.3 ng/mL). She did not undergo any tissue iron quantification method. Starting doses of deferasirox were calculated at 10 mg/kg/day, rounded to the nearest whole tablet dose. The duration of therapy varied from two to 16 months. In two patients, it was disrupted due to poor compliance (gastrointestinal discomfort), while in the remaining two, deferasirox led to a substantial decrease in ferritin concentrations. The medicine was discontinued once SF dropped below 500 ng/mL, following drug instructions.

## Discussion

Approximately 97% of children with leukemia become anemic during chemotherapy and are almost exclusively treated with blood transfusions, resulting in exogenous iron accumulation [21]. TRIO is known, yet still an underrecognized treatment complication in patients and survivors of childhood malignancies.

Regardless of ferritin serving as an intracellular reservoir for iron, its serum levels adequately reflect total iron storage pools. Determining SF is inexpensive, widely accessible, and easily reproducible, making it almost ideal to track total iron body stock [12,17]. The downside of SF as a biomarker of IO lies in its low specificity. In malignancies, initially elevated SF concentrations result from the uncontrolled inflammation and increased tumor synthesis [20]. A satisfactory response to treatment should therefore lead to normalization of SF. However, in our study, SF levels obtained at the end of the intensive chemotherapy phase (T2) were significantly higher compared to baseline values (T1), even though remission was achieved in almost all cases. This increase in ferritin is in accordance with previous research conducted by Sawicka-Zukowska et al., while 40% of children with ALL entered therapy having significant hyperferritinemia, 80% finished the treatment with SF >500 ng/mL. The finding is attributed to excessive iron influx following repeated transfusions [1]. In our analysis, 96% of subjects (in whom ferritin was measured) had SF concentrations above 500 ng/mL upon finishing the intensive treatment phase (T2). Even though mean SF values decreased during the FU (T4), a substantial percentage of survivors (60%) had significant hyperferritinemia, even months after the treatment ended. The noteworthy proportion of children at risk of iron toxicities following ALL and LL treatment regimens supports the need for ferritin tracking. However, roughly one-third, one-half, and one-fifth of participants lacked ferritin levels at the end of the intensive chemotherapy phase (T2), maintenance treatment (T3), and during the FU (T4), respectively, which indicates the lack of a standardized IO monitoring in our institution, even at the cheap and easily reproducible biochemical level. The problem of poor TRIO screening, unfortunately, goes beyond our department. Another Northern American study on children with acute leukemia, published in 2021, also reported inadequate TRIO monitoring - only 68% of participants were screened via ferritin levels [8].

Acute leukemia is more strongly linked to the development of TRIO, compared to other pediatric malignancies [11]. HR disease poses an even greater risk for iatrogenic iron tissue deposition. The most aggressive forms of myelosuppressive treatment warrant maximal transfusion loads [3,5] - a fact confirmed in our group. HR ALL patients received more than double the number of RBC units compared to SR and IR patients combined. When investigating all participants, regardless of leukemia risk or lymphoma stage, we registered that almost half of the patients received 10 or more units of RBCs, and 40% received at least 100 mL of RBCs per kg. Both parameters were previously identified as risk factors for TRIO [3-8]. The remarkable portion of patients burdened with a significant transfusion load results most likely from protocols’ intensification, inducing bone marrow toxicity. We highlight the importance of a restrictive strategy concerning RBC transfusions. Rigorous adherence to guidelines for anemia supportive care lowers the risk of unwanted iron surplus. We also advise regular tracking of the total number of RBC units administered, as it may be the most sensitive predictor of organ iron load [4]. This practice could timely identify individuals at an especially increased risk of TRIO, suitable for additional work-up (MRI), despite SF concentrations.

Our research demonstrated that patients six and older were at a higher risk of TRIO. Younger age is believed to be protective as the growth spurt causes iron to be used in cells, rather than stored [1,4,6,9,11].

Hepatic and cardiac MRI scans accurately depict tissue iron content. Invasive liver biopsy is restricted only to exceptional cases where histology contributes to management [17]. According to the International Late Effects of Childhood Cancer Guideline Harmonization Group (IGHG) recommendations for TRIO surveillance, survivors of childhood cancer who underwent hematopoietic stem cell transplantation (HSCT) and/or multiple RBCTs (exact number not specified) should further proceed to T2* MRI for liver iron quantification, in cases of repeatedly elevated ferritin (SF >500 ng/mL) [18]. Despite a considerable proportion of patients at risk of TRIO in our cohort, only four underwent MRI for tissue iron evaluation. This probably resulted from the fact that MRI is not as accessible as the ferritin assay. Secondly, we evaluated patients from 2018 to 2023, while the above-mentioned IGHG recommendations appeared in 2021. Lastly, the guidelines are tailored for long-term FU, while therapeutic protocols do not address the question of TRIO during the active treatment. To mention, two of the patients in whom MRI was done were treated for HR malignancies, including HSCT. Pediatric HSCT recipients are known to have a remarkable risk of post-HSCT TRIO, resulting from multiple factors such as repeated RBCTs, persistent dyserythropoiesis, and increased enteral absorption of oral iron due to mucositis [11,22]. The other two patients, both teenage boys, underwent hepatic and cardiac MRI only upon accidental revelation of cerebral hemosiderosis. Brain MRI scans in these cases were done for unrelated diagnostic purposes (steroid-induced psychosis and somnolence due to dehydration and electrolyte imbalance), but revealed typical signs of choroid plexus iron deposits - low signal intensity in gradient-echo sequences [23,24]. Although rare, there have been reports of transfusion-derived central nervous system IO, particularly involving the pituitary gland and choroid plexus. The latter is hypothesized to protect neurons from iron damage through a buffering mechanism, but the condition, choroid hemosiderosis, is generally perceived as asymptomatic [23]. The fact that other patients with high transfusion loads, significant hyperferritinemia, or HSCT history did not undergo the same extensive work-up (MRI) outlines once again the struggle clinicians are facing when assessing secondary IO in children with hematological malignancies.

The same may be concluded for genetic testing - there was no consensus on choosing individuals suitable for the hemochromatosis assay. Alterations (H63D heterozygosity) were detected in three out of five patients tested, and all three of them had severely elevated SF at FU (T4). Some evidence implies that H63D *HFE* mutations tend to aggravate IO in beta-thalassemia carriers, suggesting their modulating effect on iron load [25]. The correlation between IO and HH gene status was not found among childhood cancer survivors, but the conclusion was reached in a small-sample study [12], and the matter warrants further investigation. If, however, *HFE* mutations do play a role in secondary IO, baseline genetic testing could be useful in identifying children at an increased risk of TRIO and lead to an even more rigorous transfusion strategy in this population. 

Iron chelation involves parenteral or oral administration of chelators, used to remove excessive iron. All three available chelators (deferasirox, deferoxamine, deferiprone) are equally effective [26,27]. Data on the safety and use of chelators in children is limited to patients aged two and older. Guidelines for iron-loading anemias advise using deferoxamine as the first choice in children [17], but due to parenteral administration, therapy is often hampered by poor compliance [13]. Acute heart failure resulting from iron accumulation is an emergency condition for the use of chelators, and a combination of oral deferiprone and intravenous deferoxamine is recommended [28]. Deferasirox or deferoxamine are better choice for liver IO. A possible side effect when using deferiprone is agranulocytosis [29]. All patients undergoing chelation therapy should be monitored with complete blood counts, kidney and liver function tests. Knowledge on the use of chelating agents in pediatric patients and survivors of childhood cancer is minimal. Our scarce experience with chelators confirmed the inexistence of a TRIO management algorithm (no consistent indication for treatment initiation) and, unfortunately, poor adherence to deferasirox due to gastrointestinal discomfort. Deferasirox, in the form of dispersible tablets, was used as the drug of choice since it is practical for administration and does not require approval of the institution’s medical commission, assuring less paperwork load for clinicians. However, mucositis, inappetence, and vomiting complicate its use during intensive chemotherapy, and so does the frequently impaired renal function.

The major limitation of the study is its retrospective model, causing a fair amount of missed data, but the analysis provides a solid groundwork for further research on this important topic. The results from the single-center study would benefit future validation on multicenter cohorts and longer FU period, as well as a prospective design generating statistically strong data on risk factors, treatment, and outcomes. Moreover, we used ferritin as the sole biochemical marker. Prospectively, tracking C-reactive protein alongside SF would enable a more critical approach in differentiating IO from inflammatory hyperferritinemia. Adding other variables to the research, such as detecting endocrinopathies, liver and heart function, in relation to IO, would be of great clinical value. This information could lead to forming treatment recommendations for these particularly vulnerable patients. 

## Conclusions

The current study represents a single tertiary care center’s experience with TRIO in children treated for ALL and LL. We have demonstrated a high occurrence, but an unsatisfactory diagnostic approach to this important antineoplastic treatment complication. No regular iron status monitoring is routinely performed in many pediatric oncology centers, including ours, even at the cheap and easily reproducible biochemical level - SF measurement. This leads to the conclusion that TRIO is often overlooked and underdiagnosed. Older children and those with HR disease have a greater chance of developing TRIO. Transfusion burden significantly impacts iron tissue deposition. Clinicians must follow a restrictive transfusion strategy, and the number of RBC units transfused throughout treatment should be tracked to identify patients with particularly worrisome iron influx. Our data showed nonadherence to the IGHG TRIO surveillance recommendations for imaging to quantify tissue iron, with only four patients undergoing targeted MRI. Unlike the ferritin assay, MRI is neither cheap nor accessible, plus the need for sedation/anesthesia in younger patients presumably leads to underutilization of the method.

Even though most morbidities caused by TRIO can be prevented or reversed, currently, there is no treatment algorithm available for pediatric oncological patients or survivors. Therapeutic management depends on the decision of the leading clinician, as seen in our experience. Future research is needed to define safety, efficacy, ideal route of administration, and timing of chelating therapy, especially regarding different stages of chemotherapy and numerous other morbidities complicating the use of iron chelators in patients and survivors of ALL and LL.
